# Supplementary material for: PKCβ Facilitates Leukemogenesis in Chronic Lymphocytic Leukaemia by Promoting Constitutive BCR-Mediated Signalling
Source: Cancers (Basel). 2022 Dec 6;14(23):6006. doi: 10.3390/cancers14236006 (PMC9735720; doi:10.3390/cancers14236006)
Supplement: Supplementary file 1 [file cancers-14-06006-s001.zip › cancers-1984579-supplementary.pdf]

cancers

MDPI

Supplementary Material: PKCβ Facilitates Leukemogenesis in Chronic Lymphocytic Leukaemia by Promoting Constitutive BCR-Mediated Signalling

Jodie Hay, Anuradha Tarafdar, Ailsa K. Holroyd, Hothri A. Moka, Karen M. Dunn, Alzahra Alshayeb, Bryony H. Lloyd, Jennifer Cassels, Natasha Malik, Ashfia F. Khan, IengFong Sou, Jamie Lees, Hassan N. B. Almuhanna, Nagesh Kalakonda, Joseph R. Slupsky and Alison M. Michie

Supplementary Table S1. CLL patient clinical characteristics.

CLL ID	Treatment[a]	Sex	Binet Stage	ZAP-70 status[b]	Cytogenetics
85	Yes	F	A	ND	del(11q)
86	No	F	A	pos	del(11q)
91	Yes	M	C	pos	del(11q)
93	Yes	M	C	pos	del(17p)
102	Yes	F	C	pos	no del(11q) or del(17p)
113	Yes	F	C	high	del(17p)
144	No	M	B	low	del(17p)
148	Yes	M	B	low	del(11q)
150	No	M	A	high	no del(11q) or del(17p)
151	No	M	B	ND	del(11q)

[a] If previously undergone treatment, it was not within three months of sample collection. [b] ZAP-70 analysis was conducted by immunohistochemistry in the regional haematology laboratory. ND – not determined.

Supplementary Table S2. Flow Cytometry Antibodies.

Name	Conjugation	Clone	Company
**CD45**	Per-CP	30-F11	BDBiosciences
**CD19**	APC-Cy7	1D3	BDBiosciences
**B220**	PE	RA3-6132	BDBiosciences
**CD5**	APC	53-7.3	BDBiosciences
**CD5**	BV510	53-7.3	BioLegend
**CD23**	PE-Cy7	B3B4	BioLegend
**CXCR4**	BV510	2B11	BDBiosciences
**CD49d**	AF647	R1-2	BioLegend
**CD38**	APC-FIRE	90	BioLegend
**CD38**	PE	90	BDBiosciences
**BTK^{Y223}**	AF647	N35-86	BDBiosciences
**BTK^{Y551}**	AF647	24a/BTK	BDBiosciences
**Annexin V**	APC	N/A	BDBiosciences

Supplementary Table S3. PCR primer sequences.

Gene	Forward sequence	Reverse sequence
Btk	cac cag aaa gac aga ttc cg	cca tag cat tct tgg ctg tc
Egr1	gag atg atg ctg ctg agc aa	gtc gtt tgg ctg gga taa ct
Tbp	gta ccc ttc acc aat gac tc	cag cca aga ttc acg gta ga

All sequences are shown 5′ - 3″.

Supplementary Table S4. ChIP primer sequences for the Sp1 binding sites on the mouse *prkcb* promotor region.

SP1 binding sites	Forward sequence	Reverse sequence
Prkcb prom 1	gcg ttt ggt cat tgc tgg at	aca cac aca tac acg tac acc g
Prkcb prom 2	cgg tgt acg tgt atg tgt gtg t	ccc tca ttt gca tga aac cc
Prkcb prom 3	tgt ctg tgt gtg tct ctg ct	tgg tcc agc tgt gct tgg ca

Supplementary Table S5: List of antibodies used for Western blotting.

Name	Clone	Dilution	2ndary Ab	Company
**PKCα**	#2056	1:1000	Rabbit	CST
**pAKT^{S473}**	D9E	1:1000	Rabbit	CST
**AKT (pan)**	C67E7	1:1000	Rabbit	CST
**pS6$^{S235/S236}$**	D57.2.2E	1:1000	Rabbit	CST
**S6**	54D2	1:1000	Mouse	CST
**PKCβII**	C-18	1:1000	Rabbit	Santa-Cruz
**SP1**	ab227383	1:500	Rabbit	Abcam
**LYN**	#2732	1:1000	Mouse	CST
**LCK**	28/Lck	1:1000	Mouse	BD Biosciences
**EGR1**	44D5	1:1000	Rabbit	CST
**SYK**	#2712	1:1000	Rabbit	CST
**c-MYC^{S62}**	EPR17924	1:1000	Rabbit	Abcam
**c-Myc**	#9402	1:1000	Rabbit	CST
**GAPDH**	D16H11	1:1000	Rabbit	CST
**α-mouse IgG, HRP Ab**	#7076	1:10000	Horse	CST
**α-rabbit IgG, HRP Ab**	#7074	1:10000	Goat	CST

List of antibodies (Ab) and their dilutions used in TBS-T with milk or BSA. All antibodies were purchased from Cell Signalling Technologies (CST), Santa-Cruz, BD Biosciences or Abcam.

Supplementary Table S6: Gene modulation in primary CLL cells upon treatment with mithramycin. CLL cells (1 x 10^7 cells) from 4 patient samples were purified from whole blood and then incubated ± 200 nM mithramycin**.** Following treatment, RNA was harvested from the cells, assessed for quality, and subjected to gene expression analysis using a Whole human genome (4x44K) microarray kit (Agilent Technologies). The effect of mithramycin treatment is recorded as fold change in gene expression together with the adjusted P value for the 4 patient samples analyzed. The microarray data are available at the GEO repository (GSE210348).

Gene name	Fold change	Adjusted P value
**PRKCB**	-3.70	p=0.00022
**BCL2**	-1.92	p=0.00031
**LEF1**	-1.47	p=0.00032
**BLNK**	-1.09	p=0.0020
**VEGFA**	-0.561	p=0.011
**SP1**	-0.529	P=0.041

Supplementary Table S7. Gene ontology analysis of the significantly altered genes between late co-culture PKCα-KR vs. MIEV cells identifies the most enriched group of pathways. Fold change ±1.2 and p-value <0.05.

Function	Type	Enrichment Score	Enrichment p-value	% genes in group that are present	# genes in list, in group	# genes not in list, in group	GO ID	Genes in Group
lymphocyte activation	biological process	50.6836	9.74E-23	28	115	297	46649	Chd7; Ptk2b; Itgal; Nfam1; Cdh17; Itgam; Chd7; Fcrl1; Polm; Chd7; Gpr183; Clcf1; Lyl1; Icosl; Chd7; Lat2; Chd7; Gja1; Cr2; Chd7; Chd7; Cd180; Cd86; Coro1a; Chd7; Flt3l; Spi1; Chd7; Msn; Cd74; Atad5; Notch2; Zbtb1; Hhex; Bank1; Ms4a1; St3gal; Chd7; Chd7; Itgb2; Chd7; Lcp1; Chd7; Gba; Chd7; Ptprc; Bcl3; Chd7; Lck; Chd7; Impdh1; Hdac5; Chd7; Chd7; Mpzl2; Chd7; Stat5b; Icam1; Bcl2a1; Chd7; Exo1; Ezh2; Slamf6; Chd7; Satb1; Gm1327; Gm1327; Gm1327; Irf8; Zbtb7b; Chd7; Chd7; Chd7; Plcl2; Kit; Gm1327; P2rx7; Mef2c; Nbn; Cmtm7; Lilrb4; Ptprj; Fcer1g; Ifnz; Itm2a; Egr1; Sema4a; Fgl2; Gpr18; Chd7; Nfatc1; Rhoh; Sox4; Adam17; Chd7; Klrb1c; Traf3i; Ccr6; Bcl6; Nedd4; Lig4; Atp7a; Nlrc3; Gm1327; Ptpn2; Tox; Jak3; Chd7; Dock8; Ly6d; Gapt; Fzd9; Chd7; Blnk; Rasgrp; Blm; Tpd52; Swap70; Cd2; Spib; Fyn; Tnfaip; Chd7; Msh2; Ada; Il15; Cd1d1; Il12a; Cdk6; Cd79a; Slc11a; Hdac9; Foxp1; Pik3r1; Psap; Tcf3; Cxcr5; Ercc1; Ccr9; Batf; Hmgb1; Cd19; Chd7; Gimap1
immune response	biological process	47.274	2.95E-21	19	251	1056	6955	Klrd1; Ifitm1; Ptk2b; Itgal; Hck; Nfam1; Tlr1; Cdh17; Cotl1; Ighv1-62-3; Cfp; Cd37; Tnfaip8l2; Cyba; Ighv1-72; Ltb; Tgfbr3; Yes1; Rnase6; Ang; Gpr183; Tnk2; Dab2ip; H2-Aa; Icosl; H2-DMb1; Ptpn6; Lcp2; Il4ra; Ighv1-69; Lyz2; Lat2; Ccr2; Ighv1-52; Lgals3; Prkd2; Nrros; Ifngr2; Tac4; Cst7; Cr2; H2-Ab1; Ccl9; Ighv1-55; Cd180; Tagap; Ighv1-61; Pycard; Pld4; Tnfrsf1a; Cd86; Wfdc17; Coro1a; Clec5a; Tlr6; Trav15d-1-dv6d-1; Trav15d-1-dv6d-1; H2-DMa; Lat; Pdcd1lg2; Ly86; Spi1; Cd74; Ifitm2; Atad5; Tmf1; Notch2; Ctss; Tlr9; Zbtb1; Ms4a1; St3gal1; Irf5; Nckap1l; Tnf; Skap1; Cd68; Ccdc88b; Lcp1; Slc15a3; Lta; Trem1; Myo1f; Ccl3; Ptprc; Bcl3; Nlrp3; Cybb; Ifngr1; Tlr2; Prkce; Ssc5d; Mpeg1; Tnfrsf13c; C5ar1; Rgcc; Ccr5; Ccr5; Lck; Bcl6b; Ctsh; Ccl7; Ccl6; Naip2; Casp4; Btla; Igf1r; Tlr13; H2-Eb1; Fes; P2ry14; Fcgr3; Lyn; Tyrobp; Colec12; Icam1; Bcl2a1d; Rnf125; Exo1; Lyz1; Nlrc4; Slpi; Slamf6; Skp2; Gm13276; Gm13277; Gm13275; Irf8; Pmp22; H2-Eb2; Hmgb3; Ctsl; Gsdmd; Pnp2; Plscr1; Trav15-2-dv6-2; Btk; Plcl2; Fcgr1; Kit; Gm13272; P2rx7; Siglecg; Tnfrsf11a; Clec2i; Adgre1; Stard7; Mef2c; Fcgr2b; Cd84; Hmgb3; Nbn; Dennd1b; Lilrb4a; Ccr1; Ticam2; Igkv6-15; Cd274; Fcer1g; Vav3; Thy1; Ifnz; Tnfsf10; Ifnar2; Slamf9; Itm2a; Ccr7; Naip6; Ighv1-74; Sema4a; Fgl2; H2-Oa; Trav6d-7; Trav6d-7; Erap1; Naip5; Trf; C3ar1; Spn; Pde4d; Ccl2; Il1rap; Casp1; Adam17; Trim59; Pld3; Pnp; Igkv4-74; Traf3ip2; Ccr6; Src; Bcl6; Ackr2; Clec4n; Igkv4-62; Il2ra; Igkv5-45; Nedd4; Ackr3; Apobec3; Lig4; Atp7a; Gm13271; Pstpip1; Ifi205; Igkv3-9; App; Tnfsf11; Jak3; Ighv5-12-4; Ighv1-58; Ifitm3; Cxcr2; Igkv19-93; Cnpy3; Csf1r; Gas6; Il10rb; Igkv1-117; Rel; Gapt; Ighv3-5; Ppp1r14b; Il5ra; Igkv12-44; Btnl2; Trim28; Crlf2; Chid1; Blnk; Rasgrp1; Irf3; Wfdc10; Igkv4-61; Wfdc5; Fgr; Igkv4-70; Igkv6-13; Igkv10-94; Swap70; Il27ra; Mov10; Alcam; Hmgb2; Igkv9-120; H2-Ob; Fyn; Ighv1-63; Tnfaip3; Igkv12-46; Tlr7; Msh2; Igkv4-59; Ada; Il15; Cd1d1; Alpk1; Il12a; Agbl5; Endou;

								Map3k5; Rps19; Lgr4; Igkv6-32; Ptk2; Cd79a; Igkv4-86; Slc11a1; Ccl22; Trem3; Igkv5-48; Foxp1; Igkv10-96; Gpam; Tnfaip8; Stat3; Tnfrsf14; Zc3h12a; Trim32; Cxcr5; Ercc1; Slc15a4; Samhd1; Ccr9; Batf; Hmgb1; Zap70; Aim2; Ighv13-2; Cd19; Igkv2-109; Irak4; Igkv8-21; Mfsd6; Igkv8-30
production of molecular mediator of immune response	biological process	30.7882	4.25E-14	36	46	83	2440	H2-Aa; H2-DMb1; H2-Ab1; Trav15d-1-dv6d-1; Trav15d-1-dv6d-1; H2-DMa; Atad5; Trem1; H2-Eb1; H2-Eb2; Trav15-2-dv6-2; Stard7; Igkv6-15; Slamf9; Itm2a; Fgl2; H2-Oa; Trav6d-7; Trav6d-7; Igkv4-74; Traf3ip2; Igkv4-62; Igkv5-45; Igkv3-9; Igkv19-93; Gas6; Igkv1-117; Gapt; Igkv12-44; Crlf2; Igkv4-61; Igkv4-70; Igkv6-13; Igkv10-94; Igkv9-120; H2-Ob; Igkv12-46; Igkv4-59; Il12a; Igkv6-32; Igkv4-86; Slc11a1; Trem3; Igkv5-48; Igkv10-96; Igkv2-109; Igkv8-21; Igkv8-30
leukocyte migration	biological process	22.3821	1.90E-10	26	54	150	50900	Asb2; Mmp9; Itgal; Itgam; Sell; Gpr183; Cnr2; Itga4; Ccr2; Lgals3; Ccl9; Coro1a; Msn; Pecam1; Itgb2; Nckap1l; Tnf; Cklf; Gba; Trem1; Ccl3; C5ar1; Wdr1; Ccl7; Ccl6; Arhgef5; Fcgr3; Stat5b; Icam1; Kit; Ccr1; Spp1; Fcer1g; Vav3; Slamf9; Ccr7; Itga9; Pde4d; Ccl2; Ccr6; Tnfsf11; Itgb7; Cxcr2; Dock8; Gas6; Arhgef5; Ano6; Selplg; Rps19; Ccl22; Trem3; Ninj1; S1pr1; Cxcr5; Irak4
immune effector process	biological process	19.6089	3.05E-09	20	85	331	2252	Klrd1; Ptk2b; Itgal; Cdh17; Ighv1-62-3; Cfp; Ighv1-72; Gpr183; Icosl; Ptpn6; Il4ra; Ighv1-69; Lat2; Ighv1-52; Tac4; Cr2; Ighv1-55; Cd180; Ighv1-61; Pycard; Coro1a; H2-DMa; Lat; Spi1; Cd74; Atad5; Notch2; St3gal1; Lcp1; Trem1; Myo1f; Bcl3; Prkce; Rgcc; Wdr1; Ctsh; Fcgr3; Tyrobp; Icam1; Exo1; Gm13276; Gm13277; Gm13275; Irf8; Plcl2; Fcgr1; Kit; Gm13272; Stard7; Fcgr2b; Nbn; Fcer1g; Ifnz; Slamf9; Itm2a; Ighv1-74; Sema4a; Fgl2; Traf3ip2; Ccr6; Lig4; Atp7a; Gm13271; App; Ighv5-12-4; Ighv1-58; Gapt; Ighv3-5; Crlf2; Rasgrp1; Swap70; Ighv1-63; Tnfaip3; Msh2; Ada; Il12a; Slc11a1; Trem3; Foxp1; Ercc1; Batf; Hmgb1; Ighv13-2; Cd19; Irak4
leukocyte homeostasis	biological process	16.5018	6.81E-08	33	27	56	1776	Gpr174; Gpr183; Mpl; Casp3; Coro1a; Lat; Sh2b3; Gimap3; Nckap1l; Tnfrsf13c; Lyn; Stat5b; Bcl2a1a; P2rx7; Mef2c; Lilrb4a; Ikbkb; Ccl2; Traf3ip2; Il2ra; Jak3; Gapt; Tnfaip3; Men1; Bcl2l11; Slc40a1; Slc15a4
antigen processing & presentation	biological process	11.2912	1.25E-05	27	25	69	19882	H2-Aa; H2-DMb1; Ifi30; H2-Ab1; H2-DMa; Cd74; Ctss; Gba; H2-Eb1; Fcgr3; Icam1; H2-Eb2; Ctsl; Fcgr1; Fcgr2b; Fcer1g; H2-Oa; Erap1; Rab4a; H2-Ob; Cd1d1; Ide; Slc11a1; Psap; Mfsd6
somatic diversification of immune receptors	biological process	11.0128	1.65E-05	34	16	31	2200	Polm; Icosl; Atad5; Exo1; Ezh2; Nbn; Ccr6; Lig4; Swap70; Msh2; Foxp1; Tcf3; Ercc1; Samhd1; Batf; Hmgb1
activation of immune response	biological process	9.22096	9.89E-05	18	55	253	2253	Klrd1; Nfam1; Ighv1-62-3; Cfp; Ighv1-72; Icosl; Ptpn6; Lcp2; Ighv1-69; Lat2; Ighv1-52; Prkd2; Cr2; Ighv1-55; Ighv1-61; Pycard; Tlr9; Ms4a1; Nckap1l; Skap1; Ptprc; Tlr2; C5ar1; Rgcc; Lck; Lyn; Tyrobp; Bcl2a1d; Nlrc4; Btk; Clec2i; Mef2c; Dennd1b; Fcer1g; Vav3; Thy1; Ighv1-74; C3ar1; Pde4d; Clec4n; Ifi205; Ighv5-12-4; Ighv1-58; Ighv3-5; Btnl2; Blnk; Fyn; Ighv1-63; Cd79a; Zc3h12a; Hmgb1; Zap70; Aim2; Ighv13-2; Cd19
myeloid cell homeostasis	biological process	5.20486	0.0054898	26	10	28	2262	Ampd3; Mpl; Hmox1; Sh2b3; Nckap1l; Lilrb4a; Ccl2; Traf3ip2; Bcl2l11; Slc15a4
tolerance induction	biological process	4.40428	0.0122249	36	5	9	2507	Lilr4b; Lyn; Lilrb4a; C3ar1; Tnfaip3

B cell selection	biological process	3.95864	0.0190891	50	3	3	2339	H2-Ab1; Btk; Traf3ip2
T cell selection	biological process	3.88748	0.0204969	24	8	25	45058	H2-DMa; Cd74; Ptprc; Ccr7; Spn; Il15; Cd1d1; Zap70

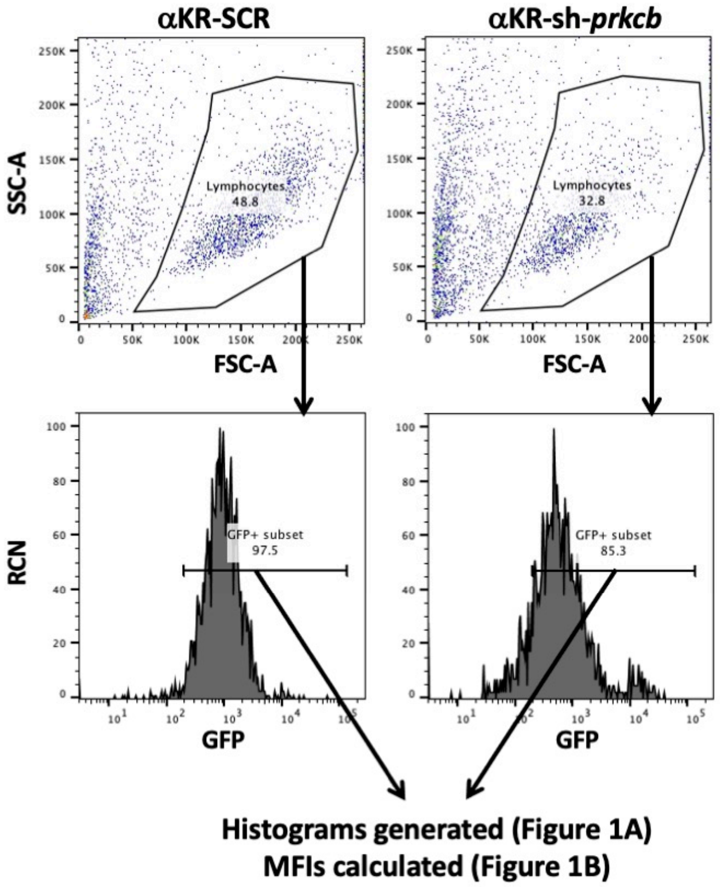

Supplementary Figure S1: Gating Strategy for cell surface marker expression. Knockdown of *prkcb* (or scrambled (SCR) control) was performed in HSPC cells within 24 hr of isolation from the mouse, then these cells were retrovirally transduced with MIEV or PKCα-KR (aKR) at d7. Cells were co-cultured with OP9 in the presence of cytokines. Phenotypic characterization of the cells was carried out by flow cytometry as shown in Figure 1A. The gated strategy for generating these data is shown here. Initially cells are live/size gated by FSC/SSC to identify the lymphocyte population (top plots). Thereafter the "Lymphocytes" were GFP+ gated and histograms (Figure 1A) and MFIs (Figure 1B) of cell surface markers were generated.

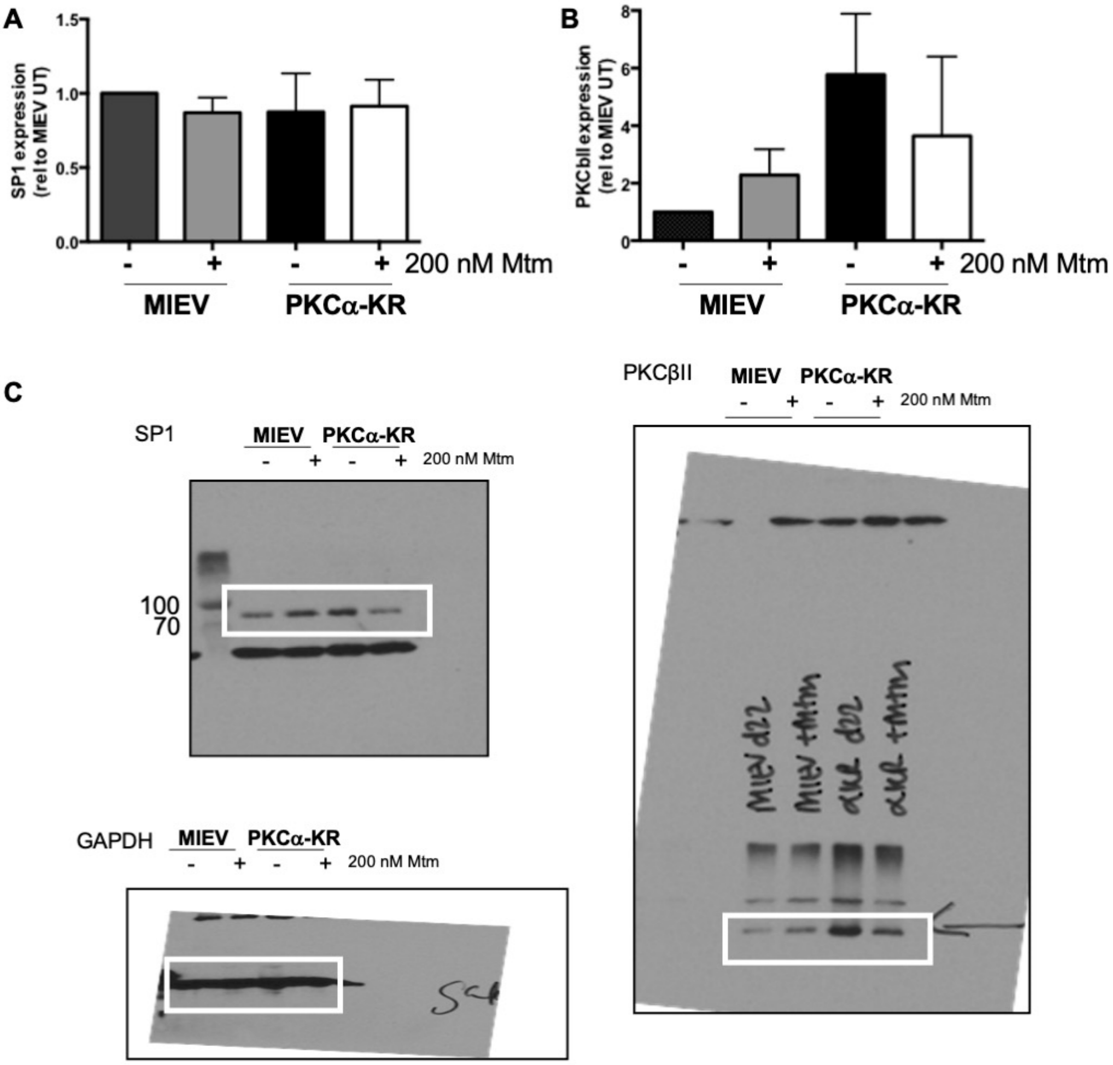

Supplementary Figure S2: Mithramycin treatment of PKCα-KR cells leads to a slight decrease in PKCβII expression. Protein lysates were prepared from MIEV and PKCα-KR cells that were treated with 200 nM mtm for 12 hr. Western blotting was performed and the resultant blots were probed for PKCβII, SP1 and a loading control (GAPDH). Densitometry was performed by normalising the target protein expression to loading control, and then expressed relative to MIEV UT sample in each blot. Densitometry of SP1 (**A**) and PKCβII (**B**) shown. n=3 individual experiments were carried out. **C.** The full blots of the representative images shown in Figure 2D.

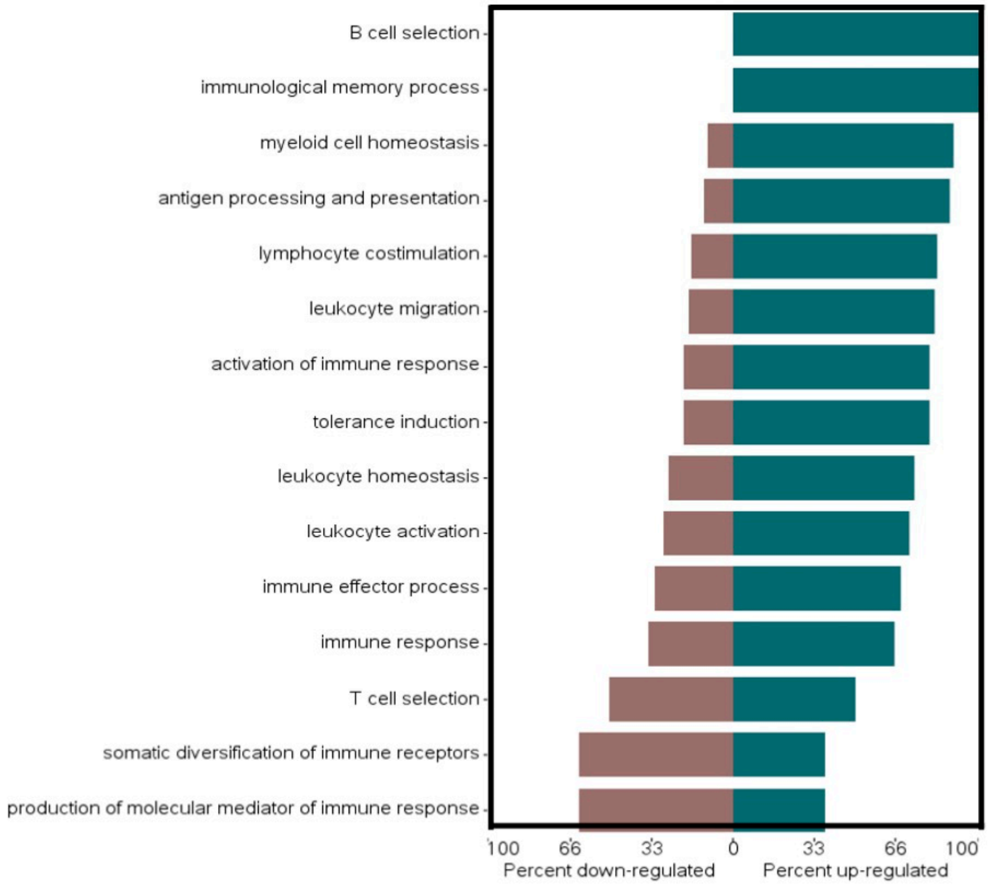

Supplementary Figure S3: Gene ontology enrichment analysis.
Gene ontology enrichment analysis identified functional groups with the highest over-representation of genes within the gene list compared to background are shown alongside their respective enrichment score (-log p-value of a chi-square test). Percentage of genes within each functional group (FDR <0.05) that are up- or down-regulated in PKCα-KR vs. MIEV cells.

A

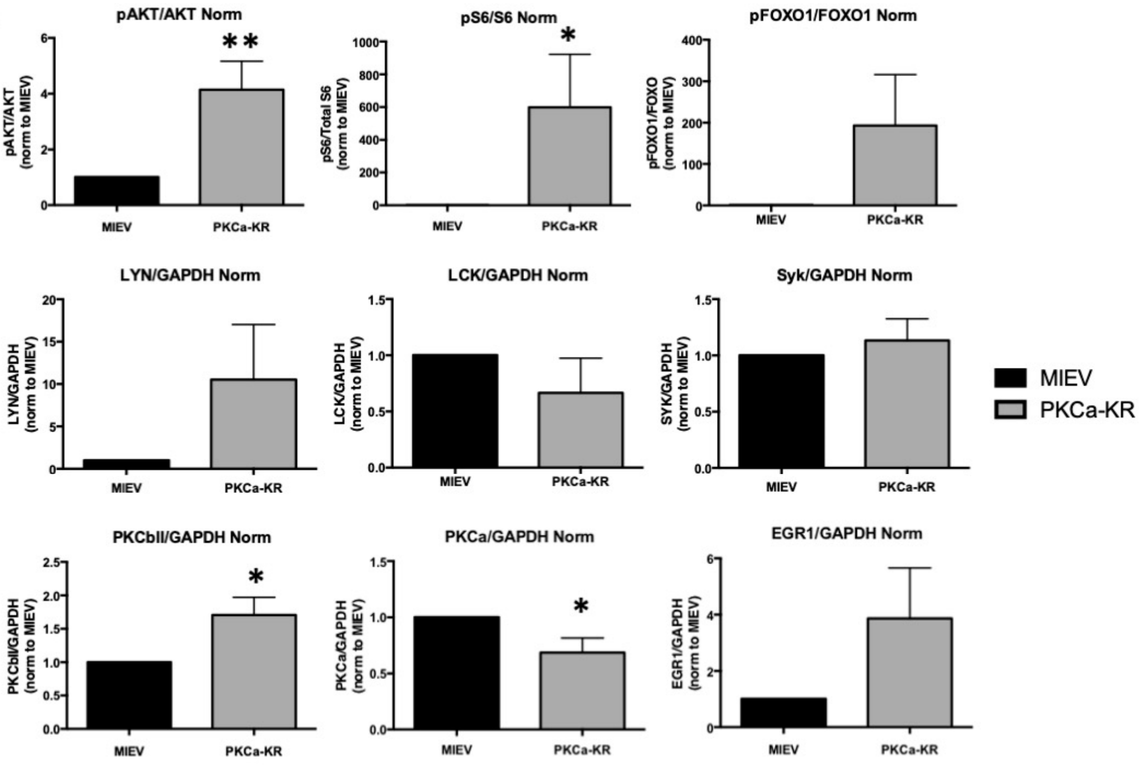

Supplementary Figure S4: PKCα-KR cells modulate BCR signaling components in a similar manner to primary CLL cells. Protein lysates were prepared from MIEV (black bars) and PKCα-KR (grey bars) cultures and Western blotting was performed.
A. Densitometry was performed by normalising the target protein expression to loading control, and then expressed relative to MIEV sample in each blot, including the images shown in Figure 4A. Unpaired student t tests were used to analyze the data (n≥3 individual samples).

B

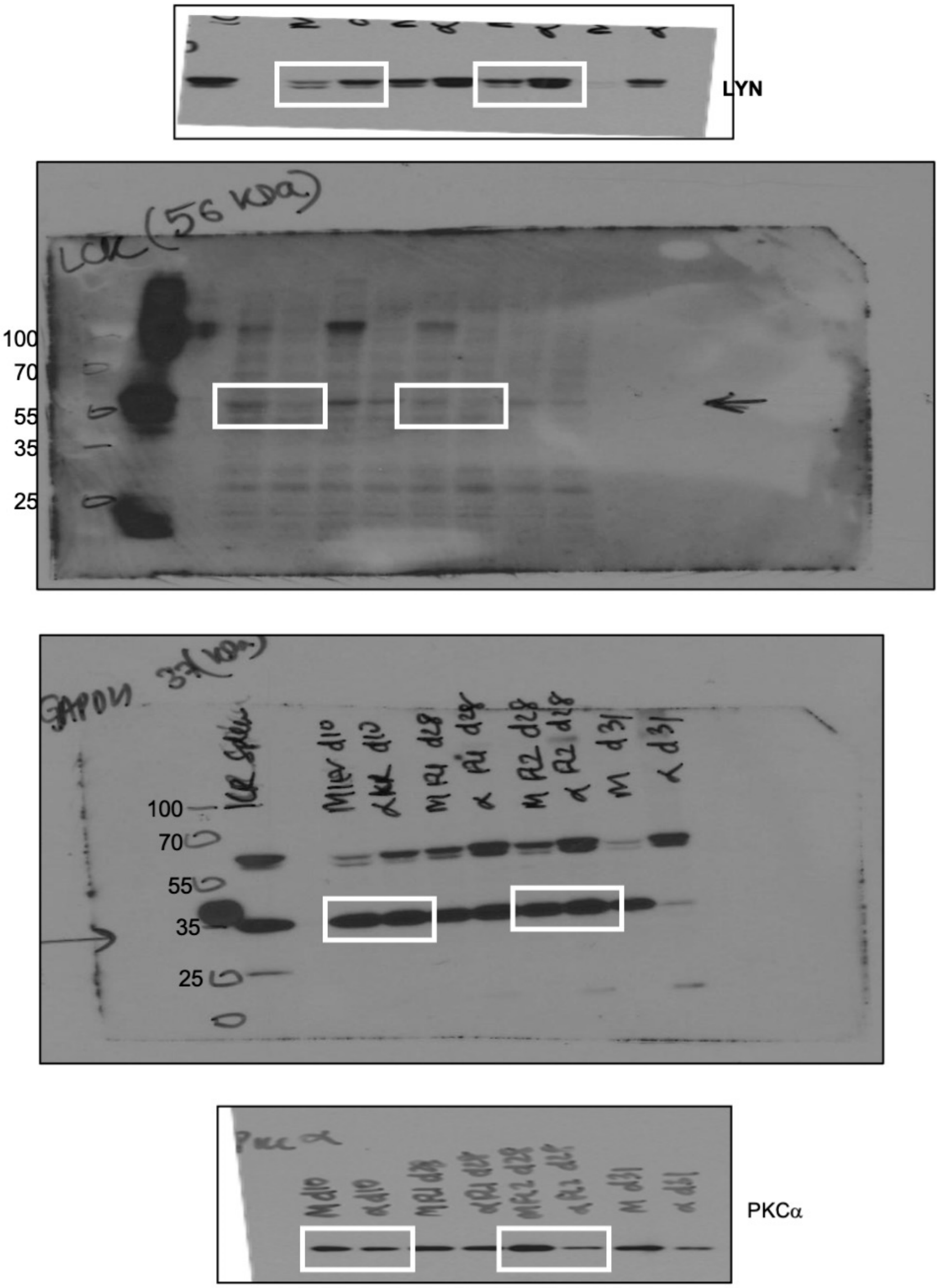

B (cont.)

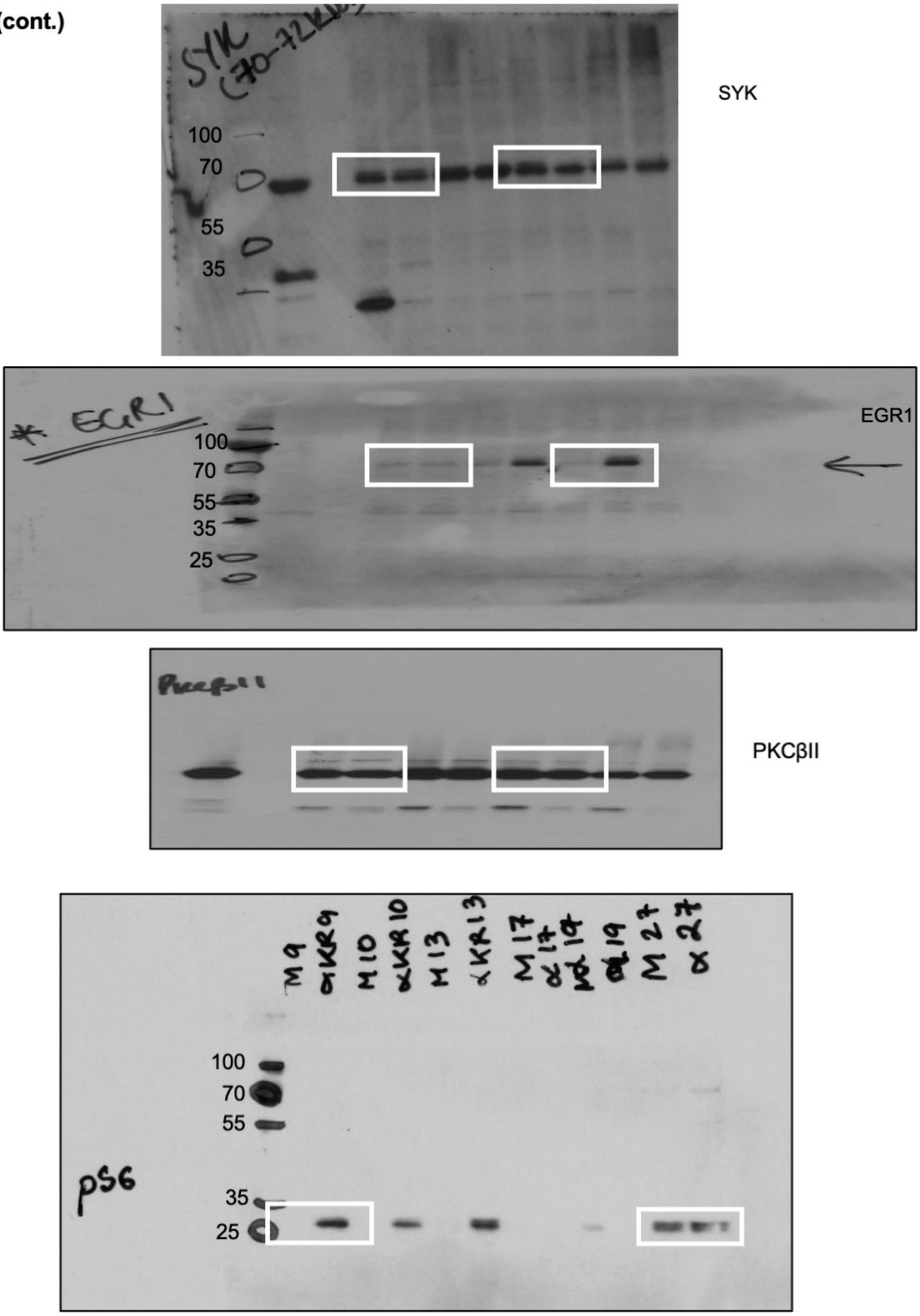

B (cont.)

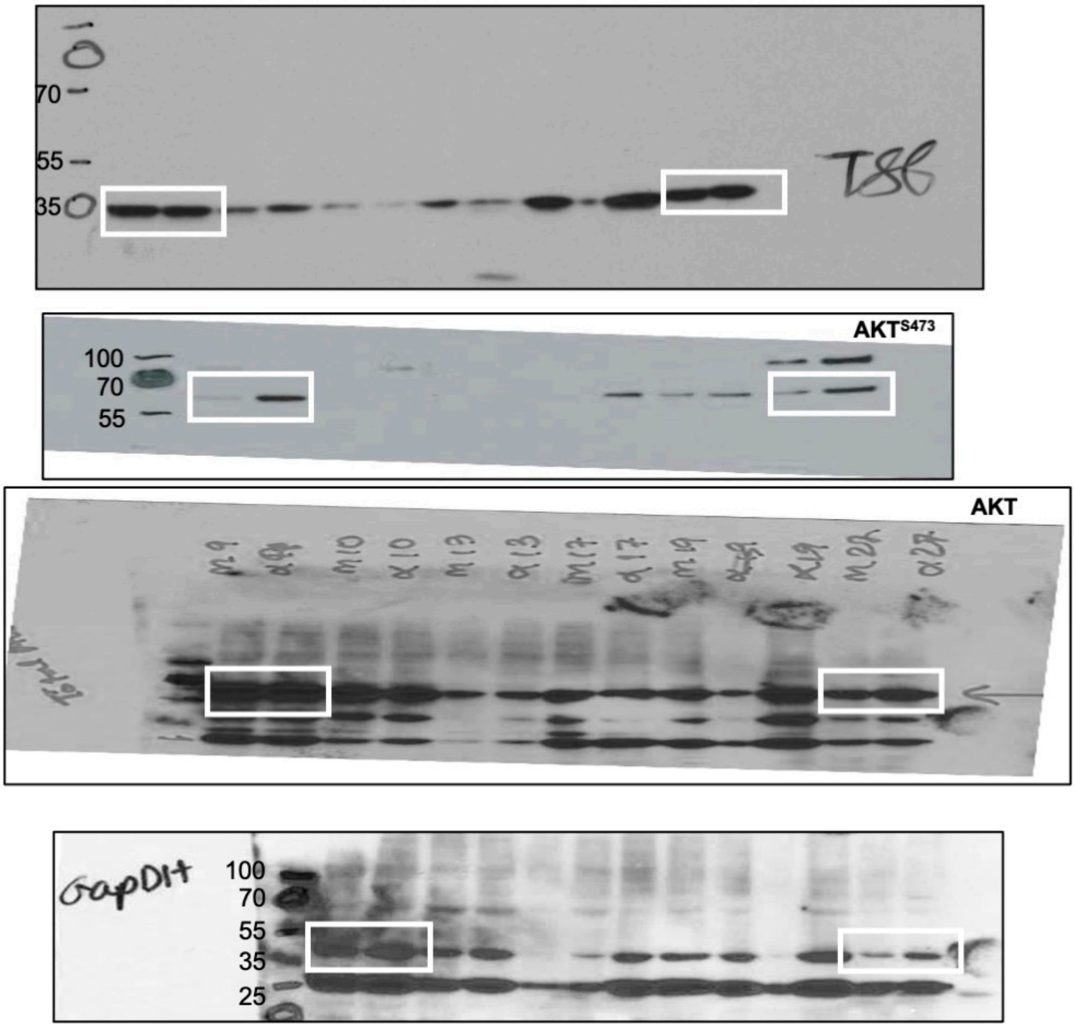

Supplementary Figure S4: PKCα-KR cells modulate BCR signaling components in a similar manner to primary CLL cells (cont). Protein lysates were prepared from MIEV (black bars) and PKCα-KR (grey bars) cultures and Western blotting was performed.
B. Mirror blots (2 – 4 gels) were run of a number of co-cultures to enable probing of the same lysates with multiple antibodies and allowing different biological replicates to be analysed on the same blots. The resultant blots were probed for the indicated target proteins and a loading control (GAPDH) on at least one of the mirror blots. Shown here are the full blots shown in Figure 4A. White boxes on the images identify the portions of the blots shown in the figure.

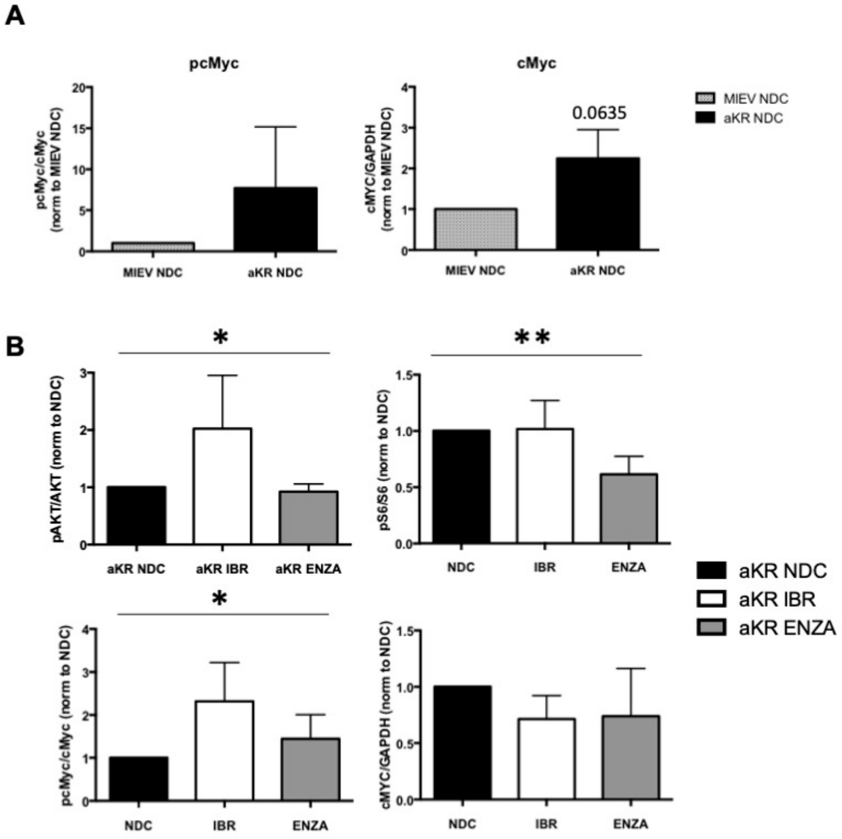

Supplementary Figure S5: Drug treatments modulate BCR signaling components in CLL-like PKCα-KR cells. MIEV and PKCα-KR cells were treated with either IB or Enza, or left NDC. Western blots were performed to identify the effect on key proteins within the BCR signalosome as indicated. Densitometry was performed by normalising the target protein expression to loading control, and then expressed relative to MIEV NDC (**A**) or PKCα-KR NDC (**B**) in each blot, including the images shown in Figure 4C. Unpaired student t tests (A) or one way ANOVA tests were used to analyze the data (n≥3 individual samples).

C

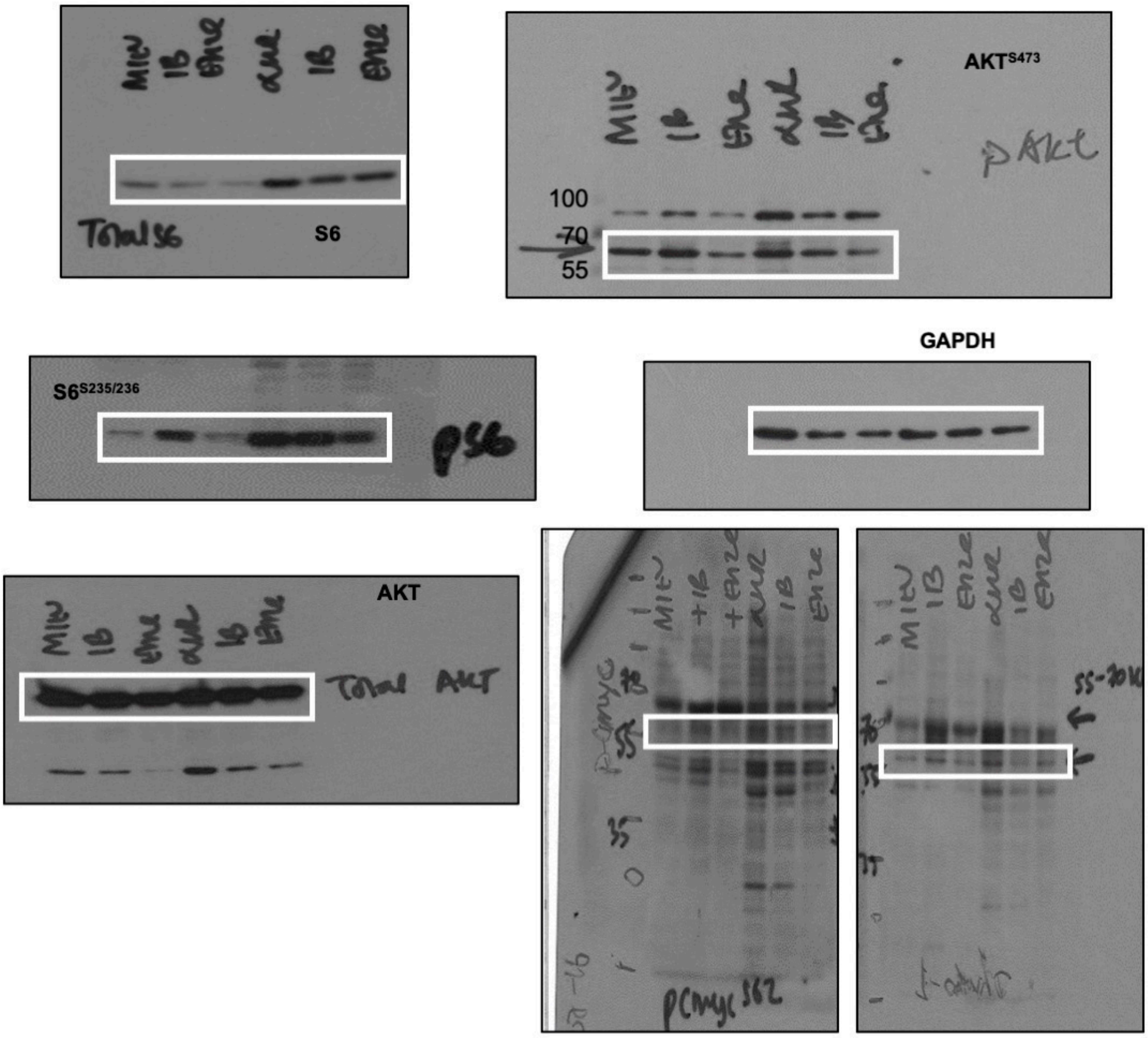

Supplementary Figure S5: Drug treatments modulate BCR signaling components in CLL-like PKCα-KR cells (cont). MIEV and PKCα-KR cells were treated with either IB or Enza, or left NDC. Western blots were performed to identify the effect on key proteins within the BCR signalosome as indicated. **C**. Mirror blots (2 – 3 gels) were loaded to enable probing of the same lysates with multiple antibodies, enabling different biological replicates to be analysed on the same blots. The resultant blots were probed for the indicated target proteins and a loading control (GAPDH) on at least one of the mirror blots. Shown here are the full blots shown in Figure 4C. White boxes on the images identify the portions of the blots shown in the figure.

A

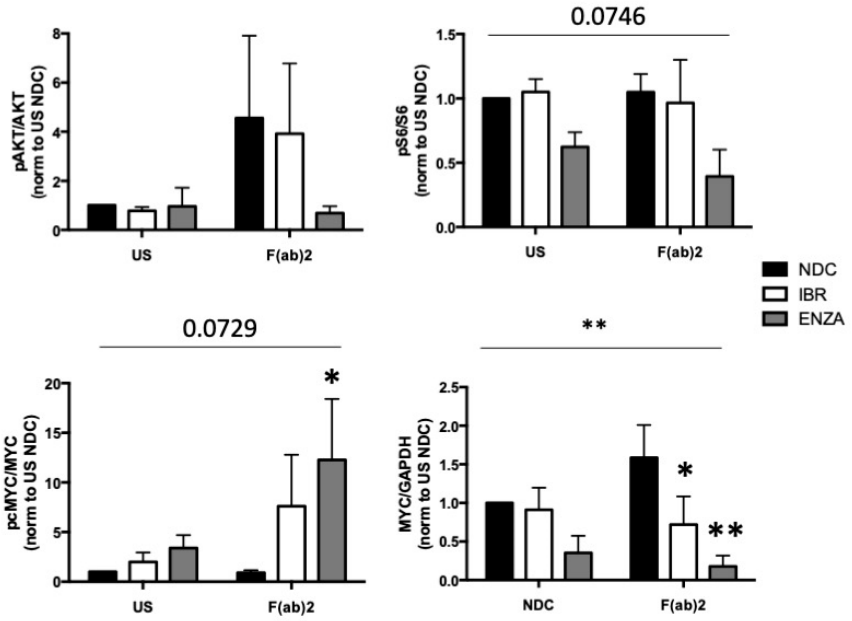

Supplementary Figure S6: Drug treatments modulate signaling components in BCR-crosslinked primary CLL samples. Human CLL cells were treated with 1 μM IB or 10 μM Enza in the presence or absence of BCR crosslinking (BCR-XL; F(ab')$_2$ fragment stimulation). Protein lysates were then prepared for Western blotting. **A**. Densitometry was performed by normalising the target protein expression to loading control, and then expressed relative to US NDC sample in each blot, including the images shown in Figure 5B. Data represents an average of n=3-6 individual patient samples. A two-way ANOVA test was used to analyze the data, with multiple comparisons.

B

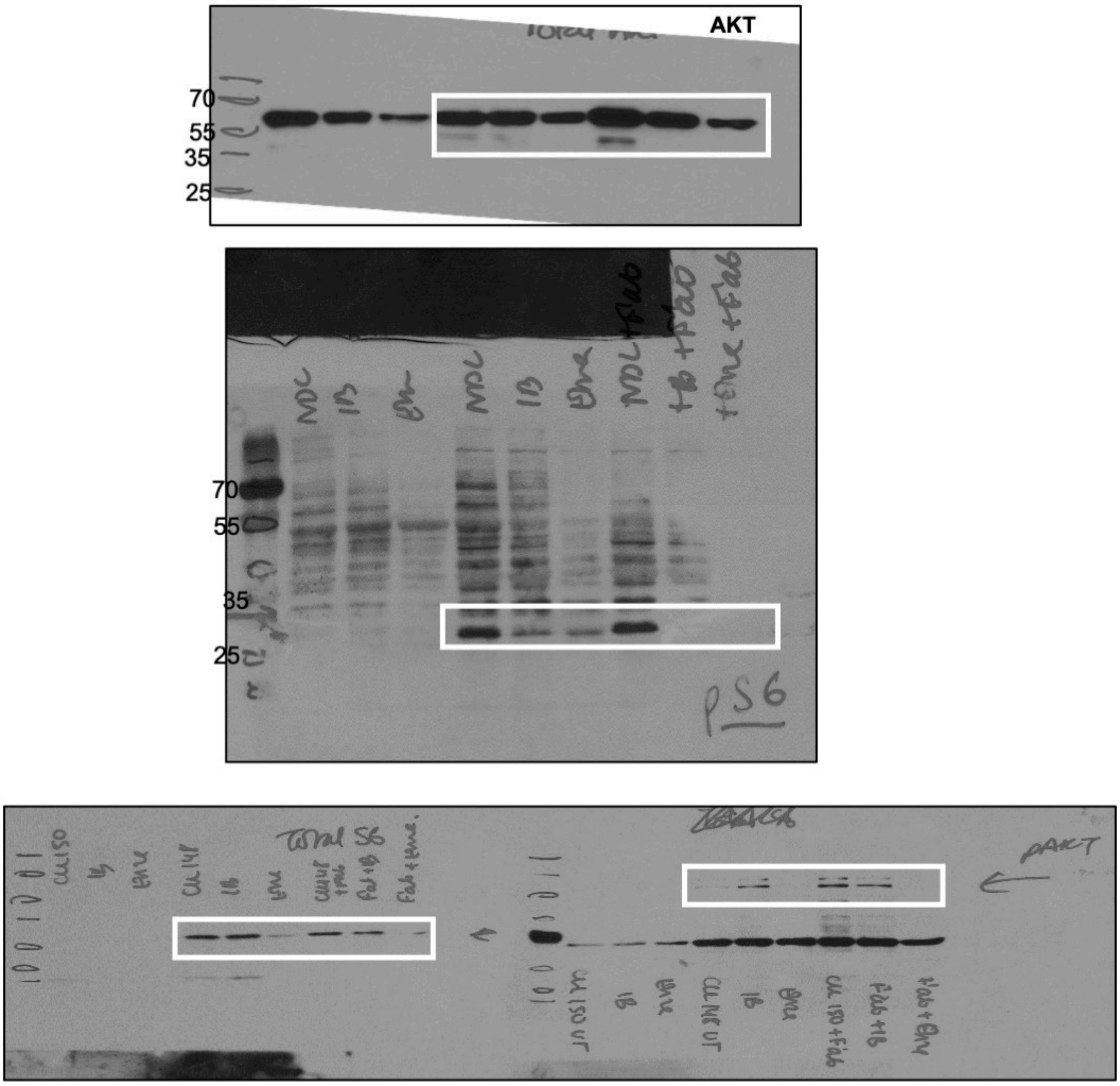

B (cont).

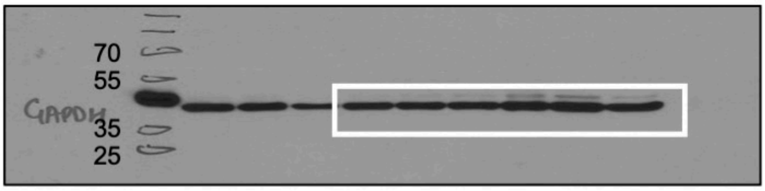

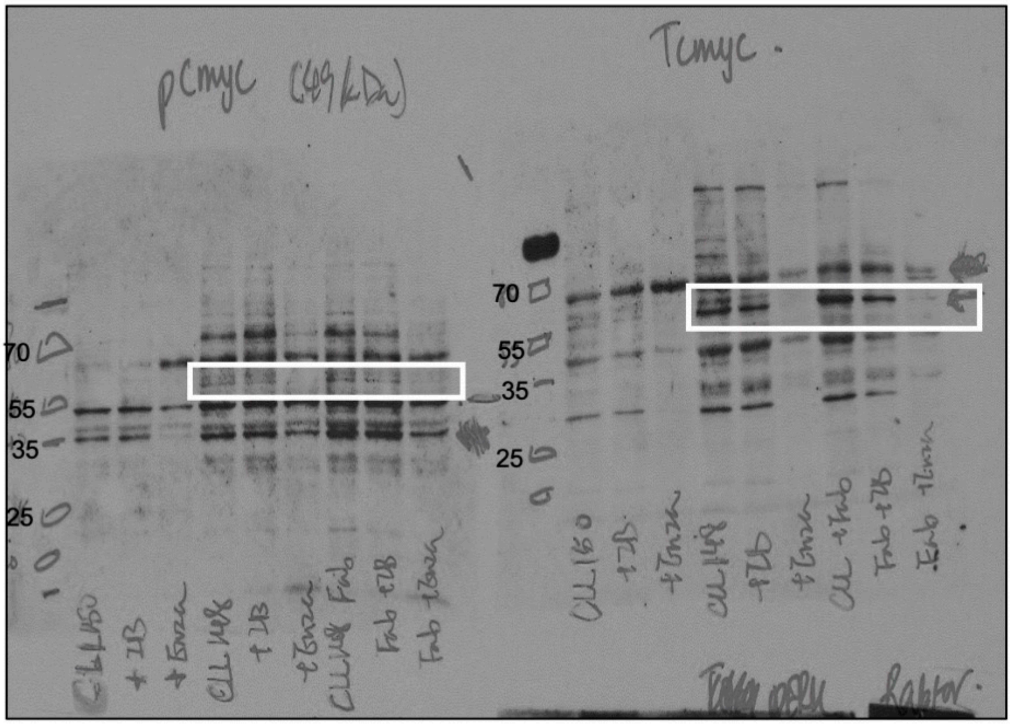

Supplementary Figure S6: Drug treatments modulate signaling components in BCR-crosslinked primary CLL samples. Human CLL cells were treated with 1 µM IB or 10 µM Enza in the presence or absence of BCR crosslinking (BCR-XL; F(ab')$_2$ fragment stimulation). Protein lysates were then prepared for Western blotting. **B**. Mirror blots (2 – 3 gels) were loaded to enable probing of the same lysates with multiple antibodies, enabling different biological replicates to be analysed on the same blots. The resultant blots were probed for the indicated target proteins and a loading control (GAPDH) on at least one of the mirror blots. Shown here are the full blots shown in Figure 5B. White boxes on the images identify the portions of the blots shown in the figure.

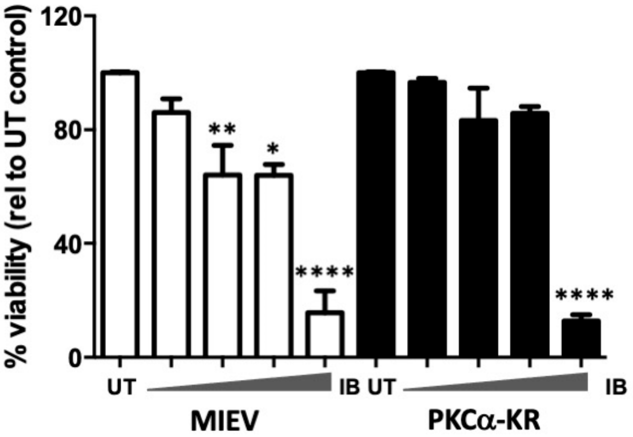

Supplementary Figure S7: The effect of ibrutinib treatment on the viability of PKCα-KR-CLL-like cells. Late co-culture MIEV and PKCα-KR cells (2 x 10^6) were cultured for 48 hr in the presence (100 nM – 10 μM) or absence (UT) of increasing concentrations of IB. 10 μM IB is above the clinically-achievable dose and effects at this concentration will likely be due to off-target effects. Viability data was generated using flow cytometry and gating on $AnnV^- 7AAD^-$ cells and expressing the percentages relative to UT cells for MIEV and PKCα-KR cultures (n≥3 individual experiments).

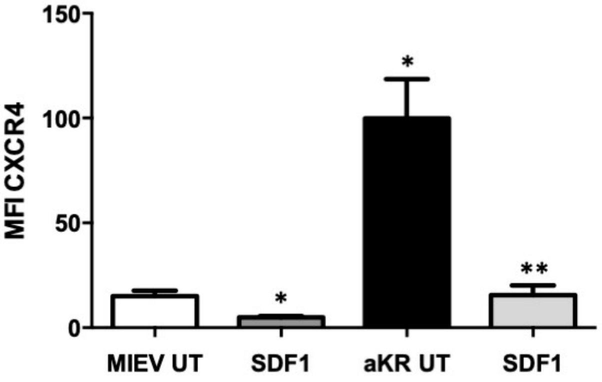

Supplementary Figure S8: Expression of CXCR4 on the surface of hematopoietic co-cultures. Late co-cultures MIEV and PKCα-KR cells (2 x 10^6) were incubated in the presence or absence of CXCL12 (SDF1) for 4 hr, replicating the conditions of the migration assay, and then stained with anti-CXCR4 antibody and analyzed by flow cytometry. Cells were live/size gated and B cells were identified by $B220^+$ expression. Results show the MFI of CXCR4 staining on cells (n≥3 individual experiments).

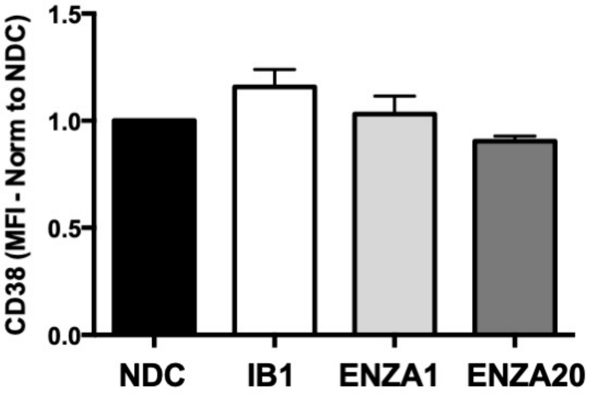

Supplementary Figure S9: The surface expression of adhesion marker CD38 was unaffected by drug treatments. Late co-culture MIEV and PKCα-KR cells (2 x 10^6) were cultured for 24 hr in the presence of 1 μM IB or 1 or 10 Enza or left untreated (NDC). The intensity of CD38 surface expression was then determined by flow cytometry. Data was live and size gated and the mean fluorescence intensity (MFI) was determined. Values are normalized to NDC as an average of three individual experiments. One way ANOVA tests were used to determine the significance of the changes in surface expression and no significant changes were found.
